# Supplementary material for: Role of a Novel Heparanase Inhibitor on the Balance between Apoptosis and Autophagy in U87 Human Glioblastoma Cells
Source: Cells. 2023 Jul 19;12(14):1891. doi: 10.3390/cells12141891 (PMC10378526; doi:10.3390/cells12141891)
Supplement: Supplementary file 1 [file cells-12-01891-s001.zip › cells-2490605-supplementary.pdf]

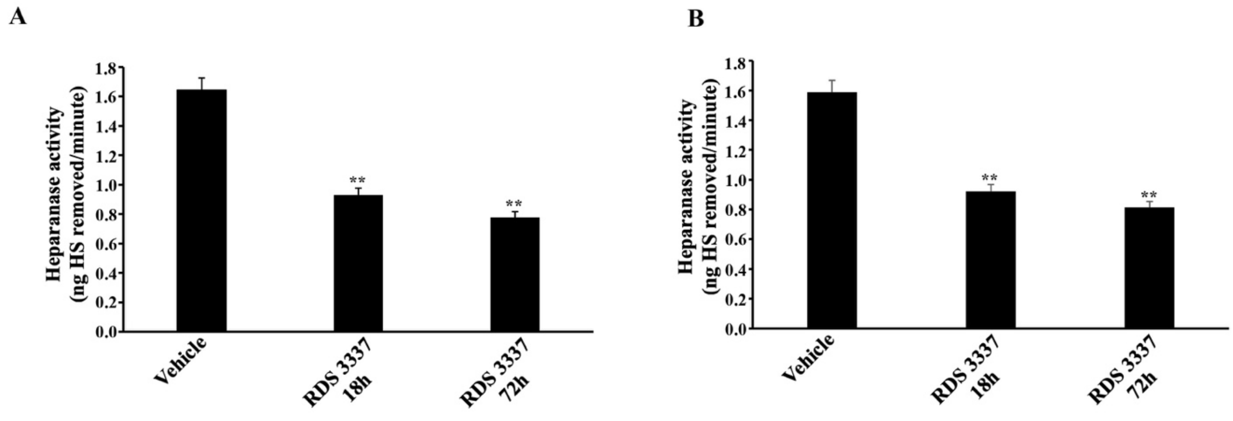

Supplementary Figure S1: Analysis of Heparanase activity. Heparanase activity was investigated in U87 (A) and in SK-N-BE2 (B) cells incubated with the benzazolyl derivative RDS 3337 at the concentration of 320 nM for 18 or 72h. After treatment, cell culture supernatants were harvested and then analyzed by an Heparanase Assay kit (Amsbio, Abingdon, UK). Columns and error bars represent the mean ± SD of 3 separate experiments. ** $p < 0.01$.

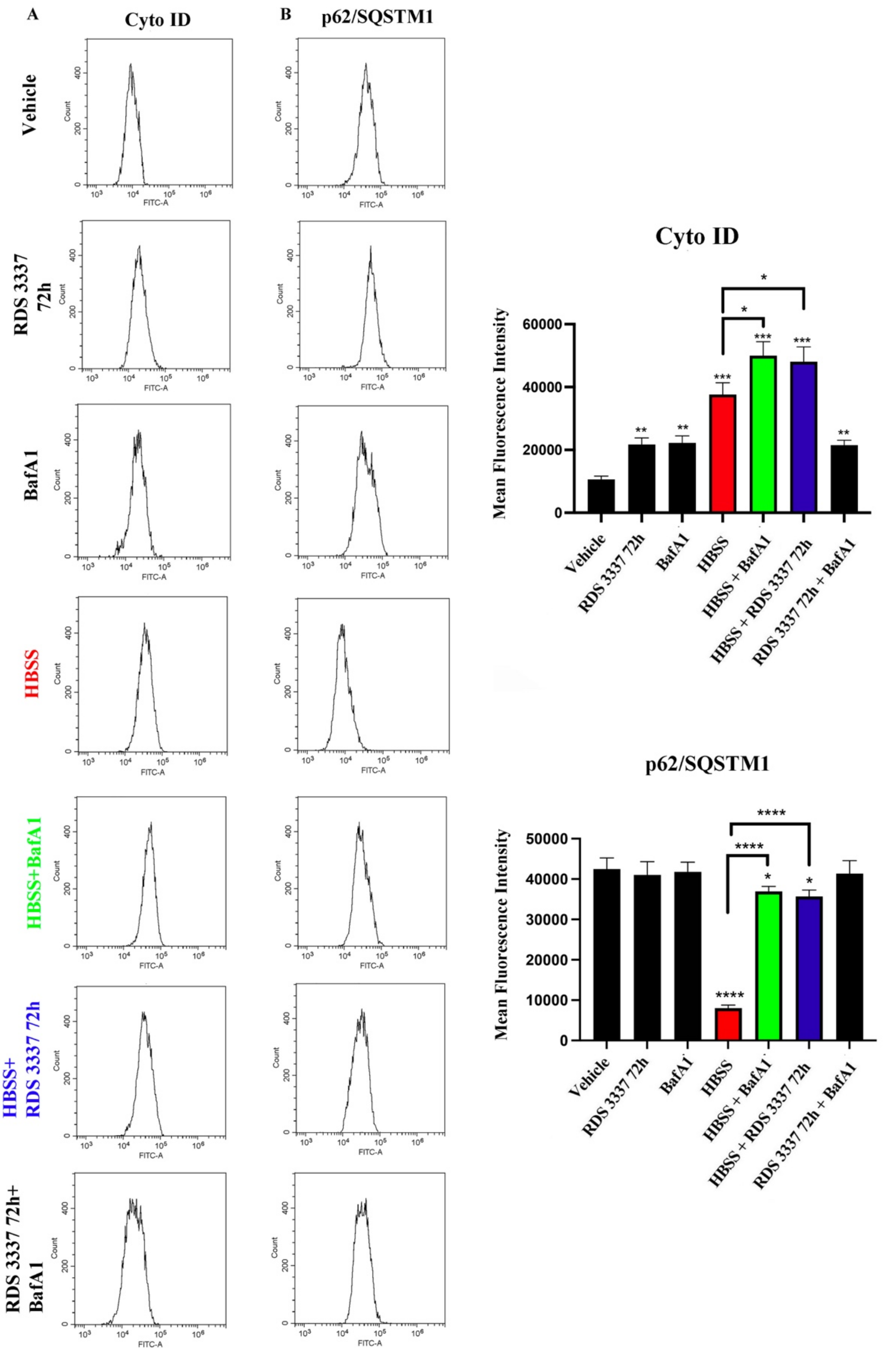

Supplementary Figure S2: Autophagy evaluation by flow cytometry in RPE-1 cells.

RPE-1 cells were starved with HBSS for 16 h, or treated with 320nM RDS3337 for 72h in the presence or absence of 100 nM Baf A1, **A)** The cells were analyzed by flow cytometry after single staining with Cyto-ID autophagy detection kit to detect autophagic vesicles (autophagosomes). **B)** p62/SQSTM1 levels were analyzed by flow cytometry with anti-p62/SQSTM1 primary antibodies followed by anti-rabbit Alexa Fluor 488. The values represent the mean ± SD of three separate experiments.* $p < 0.05$ versus vehicle,** $p < 0.005$ versus vehicle, ***$p < 0.0001$ versus vehicle, **** $p < 0.0001$ versus vehicle.

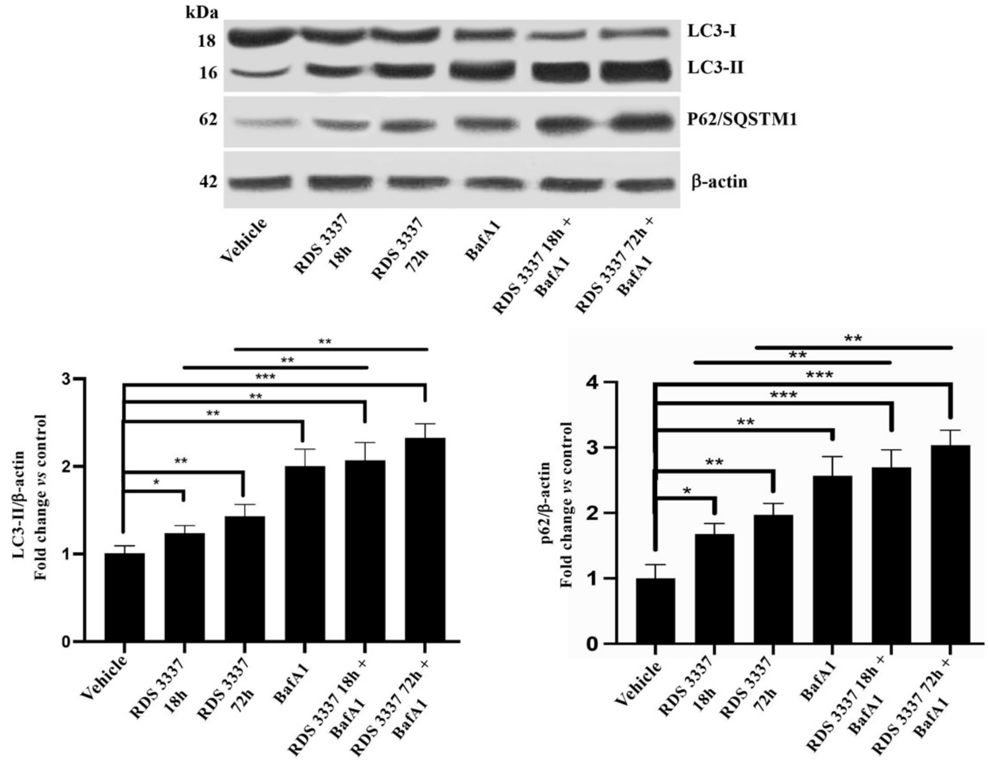

Supplementary Figure S3: RDS 3337 treatment blocks autophagic flux in SK-N-BE2 cells. Human SK-N-BE2 neuroblastoma cells, untreated or treated with 320 nM RDS 3337 for 18 or 72 h, in the presence or absence of bafilomycin A1 (Baf A1; 100 nM) were lysed in lysis buffer. The samples were analyzed for the evaluation of autophagic flux by western blot, using rabbit anti-LC3 pAb or rabbit anti-SQSTM1 mAb. Loading control was evaluated using anti-actin mAb. A representative experiment among 3 is shown. Bar graph on the right shows densitometric analysis. Results represent the mean ± SD from 3 independent experiments.* $p < 0.05$, ** $p < 0.005$, *** $p < 0.001$. No statistically significant differences were found between BafA1 and RDS 3337 18h or 72h + BafA1 samples.

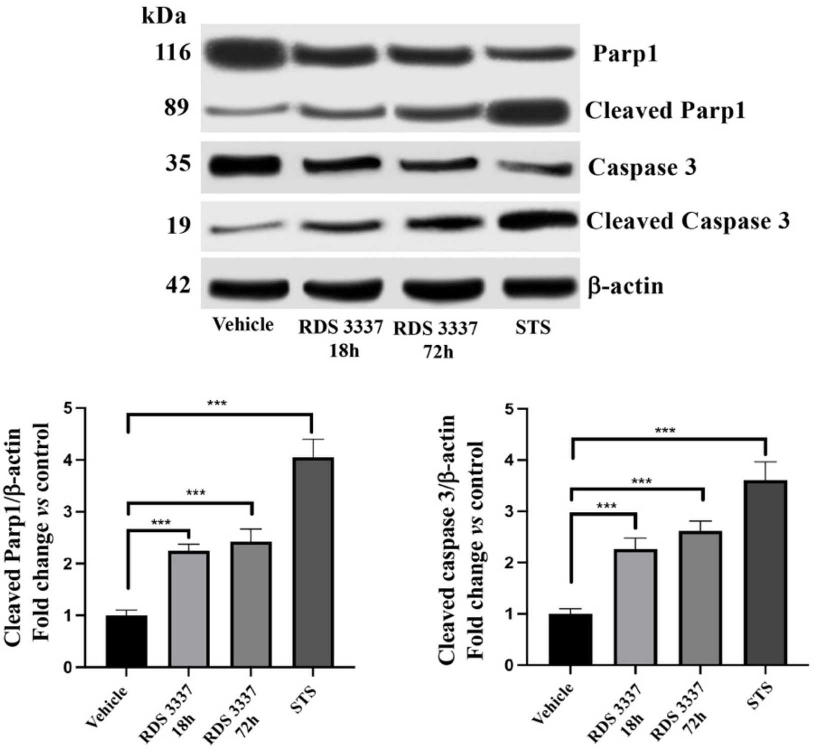

Supplementary Figure S4: RDS 3337 treatment induced a significant increase of the cleaved-caspase 3 and cleaved Parp1. SK-N-BE2 cells, untreated or treated with RDS 3337 320 nM for 18 or 72 h, were lysed in lysis buffer. The lysates were analyzed by western blot to detect caspase 3 or Parp1 levels, using anti-caspase 3 mAb or anti-Parp1 mAb. As a positive control 1 μM staurosporine (STS) was employed. Loading control was evaluated using anti-actin mAb. Bar graph on the right shows densitometric analysis. Results represent the mean ± SD from 3 independent experiments. *** $p < 0.001$.

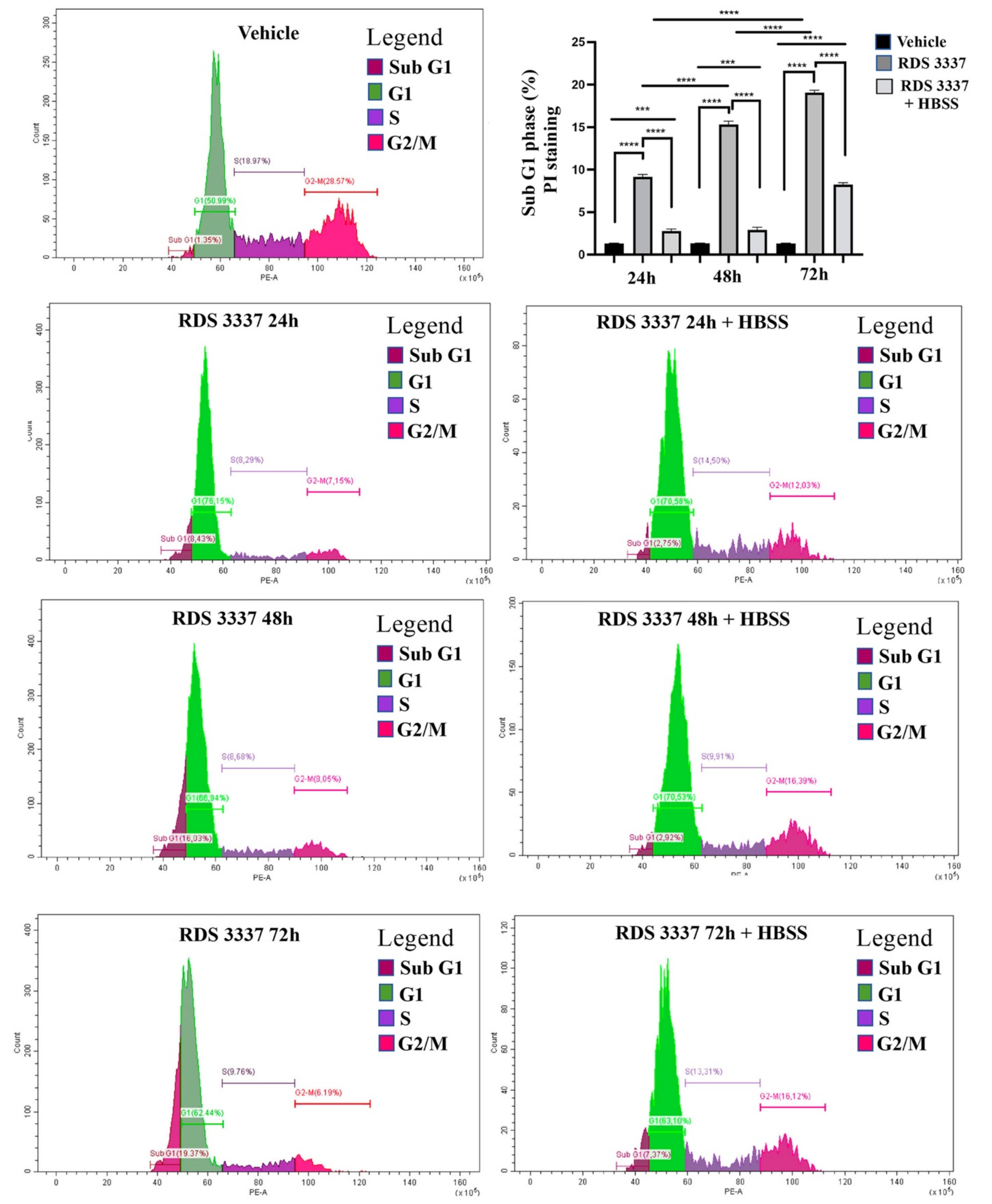

Supplementary Figure S5: RDS 3337 inhibitor sensitizes U87 human glioblastoma cells to apoptosis. U87 cells incubated with 320 nM RDS 3337 for 24, 48, and 72 h were analyzed by flow cytometric analysis after staining with propidium iodide. Alternatively, cells were incubated with RDS 3337 for 24, 48 and 72 h and then with HBSS for 16 h. Cell cycle analysis by flow cytometry subG1, G1, S, and G2/M phase in U87 cells treated with 320 nM RDS 3337. Histogram show cell cycle profiles and hypodiploid sub-G1 peak indicates typical DNA fragmentation that defines apoptosis. Columns and error bars represent the mean ± SD of three separate experiments. *** p <0.001, **** p <0.0001.

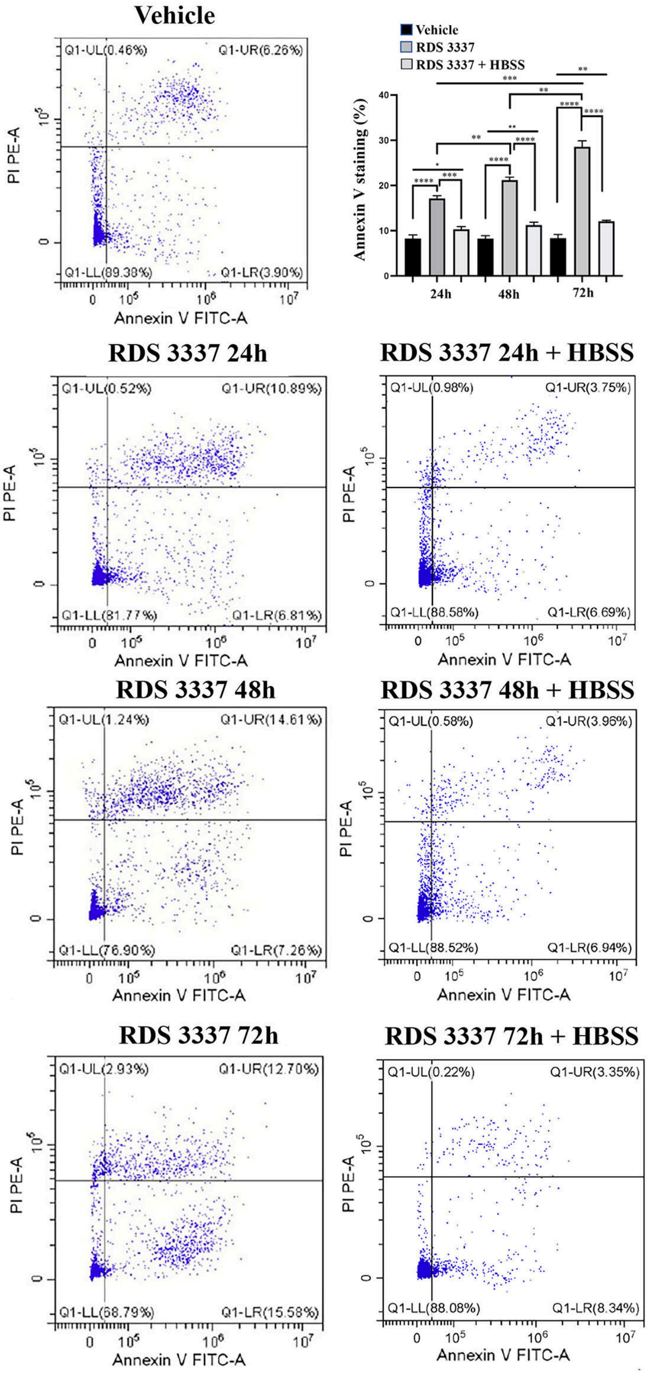

Supplementary Figure S6: Apoptosis evaluated by flow cytometry with annexin V-FITC/PI in U87 human glioblastoma cells after RDS 3337 treatment. U87 cells incubated with 320 nM RDS 3337 for 24, 48, and 72 h, or with RDS 3337 for 24, 48 or 72 h and then with HBSS for 16 h, were stained with annexin V-FITC/PI before analyzed by flow cytometry. Dot plots of propidium iodide (PI)-Annexin V by flow cytometry are showed. Bar graph shows the percentages of annexin V positive cells. The values represent the mean ± SD of three separate experiments. ** p <0.005, *** p <0.001, **** p <0.0001.
